# Reactive infectious mucocutaneous eruption secondary to parainfluenza virus

**DOI:** 10.1016/j.jdcr.2026.07.071

**Published:** 2026-08-24

**Authors:** Camille Gorena, Evan Baird, Jordan Hendrickson, Cindy E. Owen

**Affiliations:** a University of Louisville School of Medicine, Louisville, Kentucky; b Division of Internal Medicine, University of Louisville, Louisville, Kentucky; c Division of Dermatology, University of Louisville, Louisville, Kentucky

**Keywords:** MIRM, mucositis, parainfluenza, reactive infectious mucocutaneous eruption, RIME

## Introduction

Mycoplasma pneumoniae-induced rash and mucositis (MIRM) is a well-documented post-infectious mucosal disease originally proposed to differentiate it from Stevens-Johnson Syndrome (SJS) and Toxic Epidermal Necrolysis (TEN). Unlike SJS/TEN, MIRM has limited cutaneous involvement, a favorable prognosis, predominantly affects the pediatric population, and has a strong association with *Mycoplasma pneumoniae*.^1^ However, over the last decade increasing cases have demonstrated a variety of infectious agents such as *Chlamydophila pneumoniae*, enterovirus, influenza B virus, rhinovirus, HHV-7, and SARS-CoV-2.^2^, ^3^, ^4^ In response to this broadened spectrum of pathogens, the term reactive infectious mucocutaneous eruption (RIME) was proposed to more accurately characterize the disease. We report a rare case of RIME secondary to parainfluenza virus in a 42-year-old male with rapid onset of severe mucositis.

## Case summary

A 42-year-old man with a history of anxiety disorder presented to the emergency department with painful oral ulcers, eyelid erythema and edema, conjunctivitis, and odynophagia. His only medication was lamotrigine, which had been maintained at a stable dose for many years. One week prior to presentation he had rhinorrhea, a cough, and fatigue. At that time he was found to be negative for *M. pneumoniae* and *C. pneumoniae*, but positive for Parainfluenza virus upon polymerase chain reaction (PCR) testing. He was prescribed prednisone for these symptoms and denied taking any other prescribed or over-the-counter medications. Three days later, his condition worsened with the development of pharyngitis, oral ulcers, and mucosal sloughing of his lips. Nasopharyngeal endoscopy at that time revealed white lesions in the esophagus. Significant odynophagia and his inability to maintain adequate oral intake prompted his presentation to the emergency department.

Physical exam revealed bilateral conjunctival injection with discharge and eyelid edema. His lips were edematous with hemorrhagic mucosal sloughing of labial surfaces (Fig 1). Intraoral evaluation showed numerous vesicular lesions, tonsillar erythema, and a non-scrapable white plaque on the dorsal tongue. There were no lesions observed on non-mucosal skin, including targetoid or atypical targetoid lesions.

**Fig 1:**
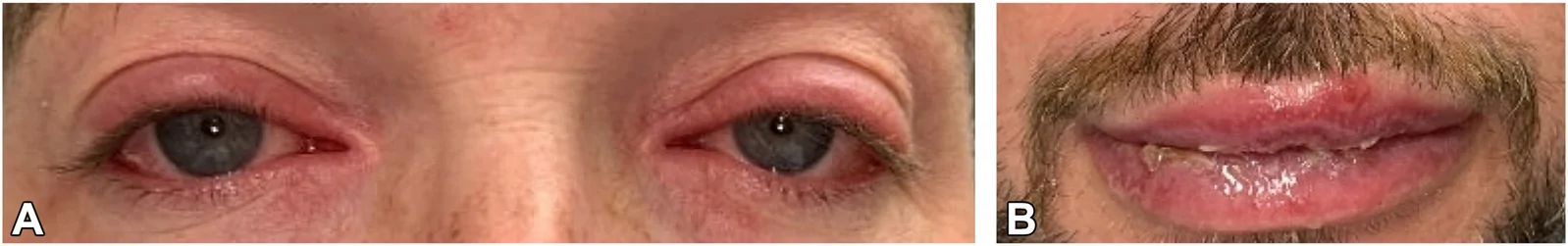
Eyelid erythema and conjunctival injection (**A**) and mucosal sloughing with hemorrhagic crusting of the labial surfaces (**B**).

Laboratory evaluation was notable for an elevated C-reactive protein (CRP) of 175.9 mg/L and was otherwise unremarkable for other inflammatory markers or metabolic profile. His respiratory panel was negative for *C. pneumoniae*, *Legionella pneumophilia*, *M. pneumoniae*, Influenza A, Influenza B, Coronavirus, and RSV. KOH preparation of the oral white plaques was also negative. Given his dermatologic presentation in the context of lamotrigine use, Stevens-Johnson Syndrome was initially considered. However, the absence of recent dose escalation or initiation, severe mucosal-limited involvement, and recent evidence of infection made RIME more likely.

The patient was initiated on intravenous fluids, analgesics, and supportive topical therapy including petroleum jelly for the lips and ocular lubricant for the eyes. On the first day of hospitalization, both ocular and oral symptoms greatly improved. On the second day of hospitalization the patient reported passage of a mucosal plug from the urethral meatus during the initiation of micturition. The emergence of genitourinary involvement provided additional support for the diagnosis. Considering treatment for RIME is mainly supportive and the patient had resumed oral intake, the patient was discharged with analgesics and prednisone and made full recovery within one week.

## Discussion

RIME has emerged as the preferred terminology for mucocutaneous eruptions caused by infectious agents. Proposed diagnostic criteria require evidence of an infectious trigger based on symptoms, imaging or lab test. Clinical presentation must also have at least 2 of the following: vesiculobullous or atypical target skin lesions affecting <10% of the body, erosive mucositis involving 2 or more mucous membranes, or a non-contributory medication history.^5^ The patient described had a prodrome 7 days prior consisting of cough, fever, and fatigue, with parainfluenza virus confirmed as the infectious trigger with PCR testing. He further met diagnostic criteria with involvement of 3 mucous membranes (ocular, oral, urethral) and no clear drug trigger. While RIME can often present with cutaneous vesiculobullous or targetoid lesions, this case broadens the clinical spectrum by highlighting that severe mucositis can occur without any cutaneous involvement.

RIME remains primarily a clinical diagnosis, but recent histologic characterizations describe a vacuolar to lichenoid interface dermatitis with apoptotic keratinocytes and cytoid bodies.^6^ In some cases, the infiltrate was neutrophil-predominate, while a case with erythema multiforme-like lesions showed MPO+/CD68+ myeloid cells.^6^^,^^7^ These findings may help exclude clinically similar conditions, however a biopsy was not performed in our patient.

All infectious testing was negative except for parainfluenza virus, indicating parainfluenza as the presumed trigger for this patient’s RIME. Human parainfluenza virus associated with RIME is not well documented. A 2017 chart review by Olson et al identified parainfluenza virus type 2 among infectious pathogens in children diagnosed with Stevens-Johnson Syndrome (SJS) or “incomplete SJS.”^8^ However, individual clinical presentations were not described per pathogen, making it difficult to determine whether the patient with parainfluenza may have more accurately represented a RIME presentation rather than true SJS.

There are no established evidence-based guidelines for treatment of RIME. It is considered a self-limiting disease, so treatment is largely supportive involving skin care, nutritional support, and pain management. About 80% of patients fully recover, with mortality around 3%.^1^ More advanced cases may require interdisciplinary treatment involving urology, ophthalmology, and otolaryngology.^9^ Systemic steroids are often used for RIME, most commonly IV methylprednisolone or oral prednisolone followed by a gradual taper. Cyclosporine has been shown to reduce hospital stays and improve symptoms.^10^ Our patient's favorable response to supportive care with intravenous fluids, analgesics, and systemic corticosteroids is consistent with the generally benign prognosis of RIME. Rapid symptom improvement and successful transition to oral intake allowed for discharge without the need for specialized consultations.

This case supports the broader conceptualization of RIME as a reactive syndrome with diverse microbial triggers. Parainfluenza virus should be considered by clinicians and dermatologists as a possible precipitant of RIME. Although RIME is most commonly documented in children, our patient illustrates it should remain on the differential for adults. Awareness of RIME in adults is critical, as misdiagnosis as SJS/TEN will lead to unnecessary and aggressive management, despite RIME’s favorable prognosis. Further investigation into the underlying immunopathogenic mechanisms may inform targeted therapeutic approaches.

## Conflicts of interest

None disclosed.
